# Functional and Psychosocial Outcomes of Hand Transplantation Compared with Prosthetic Fitting in Below-Elbow Amputees: A Multicenter Cohort Study

**DOI:** 10.1371/journal.pone.0162507

**Published:** 2016-09-02

**Authors:** Stefan Salminger, Agnes Sturma, Aidan D. Roche, Laura A. Hruby, Tatjana Paternostro-Sluga, Martin Kumnig, Marina Ninkovic, Gerhard Pierer, Stefan Schneeberger, Markus Gabl, Adam Chelmonski, Jerzy Jablecki, Oskar C. Aszmann

**Affiliations:** 1 Division of Plastic and Reconstructive Surgery, Department of Surgery, Medical University of Vienna, Vienna, Austria; 2 Christian Doppler Laboratory for Restoration of Extremity Function, Division of Plastic and Reconstructive Surgery, Department of Surgery, Medical University of Vienna, Vienna, Austria; 3 Department of Physical Medicine and Rehabilitation, Medical University of Vienna, Vienna, Austria; 4 Department of Physical Medicine and Rehabilitation, Danube Hospital Vienna, Vienna, Austria; 5 Center for Advanced Psychology in Plastic and Transplant Surgery, Department of Medical Psychology, Medical University of Innsbruck, Innsbruck, Austria; 6 Department of Physical Medicine and Rehabilitation, Medical University of Innsbruck, Innsbruck, Austria; 7 Department of Plastic, Reconstructive and Aesthetic Surgery, Medical University of Innsbruck, Innsbruck, Austria; 8 Departments of General and Transplant Surgery, Medical University of Innsbruck, Innsbruck, Austria; 9 Department of Traumatology, Medical University of Innsbruck, Innsbruck, Austria; 10 Hand Trauma Center, St. Hedwigs’s Hospital, Trzebnica, Subdepartment of Replantation of Limbs, Trzebnica, Poland; 11 State Higher Medical Professional School, Opole, Poland; University of Toledo, UNITED STATES

## Abstract

**Background:**

Hand-transplantation and improvements in the field of prostheses opened new frontiers in restoring hand function in below-elbow amputees. Both concepts aim at restoring reliable hand function, however, the indications, advantages and limitations for each treatment must be carefully considered depending on level and extent of amputation. Here we report our findings of a multi-center cohort study comparing hand function and quality-of-life of people with transplanted versus prosthetic hands.

**Methods:**

Hand function in amputees with either transplant or prostheses was tested with Action Research Arm Test (ARAT), Southampton Hand Assessment Procedure (SHAP) and the Disabilities of the Arm, Shoulder and Hand measure (DASH). Quality-of-life was compared with the Short-Form 36 (SF-36).

**Results:**

Transplanted patients (n = 5) achieved a mean ARAT score of 40.86 ± 8.07 and an average SHAP score of 75.00 ± 11.06. Prosthetic patients (n = 7) achieved a mean ARAT score of 39.00 ± 3.61 and an average SHAP score of 75.43 ± 10.81. There was no significant difference between transplanted and prosthetic hands in ARAT, SHAP or DASH. While quality-of-life metrics were equivocal for four scales of the SF-36, transplanted patients reported significantly higher scores in “role-physical” (p = 0.006), “vitality” (p = 0.008), “role-emotional” (p = 0.035) and “mental-health” (p = 0.003).

**Conclusions:**

The indications for hand transplantation or prosthetic fitting in below-elbow amputees require careful consideration. As functional outcomes were not significantly different between groups, patient’s best interests and the route of least harm should guide treatment. Due to the immunosuppressive side-effects, the indication for allotransplantation must still be restrictive, the best being bilateral amputees.

## Introduction

The loss of a hand is a devastating, life changing event. As an essential part of our bodily appearance, hands are vital to our development and psychological well-being, and play an important role in determining our professional career.[1–3] Attempts to replace this complex organ have been developing over the past 70 years in both the fields of surgery and rehabilitation.[4,5] The concurrent developments of hand transplantation and prosthetic limbs have enabled two different options for patients who have suffered limb loss.[6] Each offer their unique set of advantages and disadvantages, yet surprisingly direct comparison at a similar level of amputation has not yet been performed.[6] This is especially worthwhile as indications for each treatment must be carefully considered depending on level of amputation, profession, age and patient’s expectations.[7]

### Hand Transplantation

The first documented hand transplantation was performed in Ecuador in 1964, yet only 2 weeks later the hand was removed.[8] This was due to insufficient immunosuppressive treatment leading to rejection.[9] Improvements in immunosuppressive regimes used in solid organ transplantation encouraged further hand transplantation attempts. As a result, a group in France performed the first successful human hand transplant in a below-elbow amputee in 1998.[10] This hand remained viable for 29 months, however the patient failed to maintain the needed immunosuppressive medication leading to chronic rejection, hand malfunction and ultimately amputation.[11] Still, reconstructive transplantation has the unique potential of not only restoring the motor skills associated with the hand, but also sensation and self-perception.[12] Important issues related to hand transplantation include the limits and boundaries of medical intervention, the potential benefits of the technique, the calculation of risk and perceived risk, the medical and psychological selection, preparation, and management of patients.[13]

Between 1998 and 2014, 107 upper extremity transplantations in 72 patients have been performed and listed in the international registry on hand and composite tissue transplantation including 26 centers worldwide.[14] Of these, there are 24 known limb losses due to various reasons (non-compliance, arterial ischemia, bacterial infection and necrosis due to sepsis) and while good outcomes were reported, there were variations in the objective measurements used between centers.[14,15] The severity of the sequel of immunosuppression cannot be understated, as the latest reports including combined transplantations had to accept fatalities as a direct consequence of immunosuppression.[16] Furthermore, this procedure necessitates extensive hand therapy resulting in long recovery and hospitalization.[17]

### Prosthetic Fitting

Prostheses conversely offer the ability to restore hand function without the risk of immunosuppression. Myoelectric prostheses have increasingly advanced ergonomic and functional features.[18] As such, prosthetic fitting with myoelectric devices is the standard of care in upper limb amputees.[19] Controlling the prosthesis for a below-elbow amputee is mostly intuitive and easy to learn in an adequate rehabilitation setting. No additional surgery is needed to fit a below-elbow amputee with a prosthetic device, and patients can return to near normal life reasonably quickly. While the control of artificial limbs is limited by the interface between the patient and the prosthesis, increasing computing power and the ability to decode bio-signals are leading to ever more natural movements.[18,19] However, prostheses use is challenging in activities like grooming, may result in discomfort at the stump region, and devices need to be serviced regularly. This leads to abandonment of the prosthetic device in estimated 20% of upper-limb amputees.[20]

Although studies are available which have analyzed differences between replantation and prostheses, none of these examined the functional and quality of life outcomes of transplanted and prosthetic limbs together.[21–24] The need for such an investigation has been highlighted by the specialist community.[25] Presented here is a multi-center cohort study comparing the functional outcomes of patients whose hands were reconstructed either by means of transplantation or prosthetic fitting from three different centers in Austria and Poland.

## Methods

### Patients

Twelve patients who underwent either hand transplantation (n = 5, Group 1) or prosthetic fitting (n = 7, Group 2) after uni- or bilateral below-elbow amputation, and had completed rehabilitation, gave informed written consent to take part in this study. This study was approved by the local institutional review board at the Medical University of Vienna.

Exclusion criteria: patients with visual impairment; and those who were not available for functional and psychosocial assessments. The uni- (n = 3) and bilateral (n = 2) transplanted patients were from the Medical University of Innsbruck, Austria (n = 3) and the Hand Trauma Center, St. Hedwig’s Hospital Trzebnica, Poland (n = 2). Overall, approximately 10% of viable below-elbow-transplanted hands of the worldwide cohort to date were enrolled in this study.

All 7 prosthetic patients were from the Medical University of Vienna. These patients were fitted with various myoelectric prostheses (Michelangelo-Hand (n = 2), SensorHand Speed (n = 3) and Transcarpal Hand (n = 2)) depending on the exact level of amputation, all from OttoBock Healthcare Products GmbH, Vienna. All of these prostheses were controlled by simple direct control.

Altogether, 7 transplanted hands were compared with 7 prosthetic hands.

### Functional Outcome Measures

Global upper extremity function was evaluated using the Action Research Arm Test (ARAT), Southampton Hand Assessment Procedure (SHAP) and the Disabilities of the Arm, Shoulder and Hand (DASH) questionnaire, which monitor hand and extremity function closely related to activities of daily living (ADL).

The ARAT is an observational test used to determine upper-limb motor function.[26] It consists of 4 sections with different tasks and a maximum of 57 points.[26] The ARAT was performed according to the standardized approach [27] and is used by different hand transplant programs for functional evaluation.[17,28,29] The SHAP is a clinically validated hand function test and was originally developed to assess the effectiveness of upper-limb prostheses, and has also been applied for the assessment of musculoskeletal and neurological conditions.[30] It is made up of 8 abstract objects and 14 ADL with each task timed by the participants themselves. Normal hand function is regarded as 100 points.[30] Although bimanual activities are included, the other hand is only needed to stabilize objects and does not really influence the score. Thus, every hand, also in the bilateral transplanted patients, is scored by it’s own.

Bimanual tasks in daily-life were rated with the DASH, a questionnaire where a score of 100 indicates the worst and 0 indicates the best hand function.[31]

### Quality-of-Life (QOL) Assessment

Patients’ QOL and overall satisfaction with their reconstruction, was evaluated with the SF-36 Health Survey (SF-36).

The SF-36 yields an 8-scale profile of functional health and well-being scores as well as psychometrically-based physical and mental health summary measures and a preference-based health-utility-index. The usefulness of the SF-36 in estimating disease-burden and comparing disease-specific benchmarks with general population norms is illustrated in articles describing more than 200 diseases and conditions.[32] Response categories range from nominal scaling to 6-point scales.[33] The SF-36 provides a general QOL-assessment by calculating four scales each for physical and mental health.

### Statistical Analysis

Both the SHAP and SF-36 outcome assessments used categorical variables and the data between the groups was unpaired. As such, a two-tailed Mann-Whitney-U-test was used for the analysis, with an alpha level of 0.05. The null hypothesis for each test was that the mean of the population from which the samples were taken, was the same for both groups. Equality of variances was not assumed.

## Results

### Patient Demographics

All patients in Group 1 (n = 5) were male and aged 40.40 ± 10.21 years at the time of transplantation (Table 1). Limb losses were due to explosion, high-voltage or industrial accidents and amputations were on the level of the wrist (n = 2), distal forearm (n = 1) and proximal forearm (n = 2). Initially, all Innsbruck patients were fitted with prostheses for a mean time of 4.67 ± 1.53 years before transplantation was performed. In the bilateral patients, the main reason for hand transplantation was the lack of sensitivity and the known limitations of prostheses for example in wet environments. The Polish patients, did not have access to prostheses, therefore transplantation was the only option available to them. Functional assessment of all patients took place 9.00 ± 3.87 years after transplantation. Details about transplantation surgery and postoperative rehabilitation are published elsewhere.[12,34]

**Table 1:** Patient’s demographics.

	Patient	Age at amputation	Age at reconstruction	Age at investigation	Time between reconstruction and investigation in years	Side of amputation	Dominant Hand before amputation	Level of amputation	Nature of loss
**Group 1 Transplant**	1	40	46	60	14	bilateral	right	dist. forearm	Explosion injury
	2	38	41	53	12	bilateral	right	prox. forearm	Electric current accident
	3	49	54	59	5	right	right	wrist	Machine accident
	4	22	29	35	6	right	right	wrist	Machine accident
	5	24	32	40	8	right	right	prox. forearm	Machine accident
	Mean/SD	**35.86 ± 9.56**	**41.29 ± 8.60**	**51.43 ± 10.08**	**9.25 ± 3.87**				
**Group 2 Prostheses**	1	22	22	26	4	left	right	mid forearm	Electric current accident
	2	33	33	35	2	left	left	mid forarm	Climbing accident
	3	26	26	29	3	right	right	mid forarm	Motorcycle accident
	4	25	25	26	1	right	left	prox. forearm	Machine accident
	5	29	29	29	1	left	right	wrist	Machine accident
	6	28	28	37	9	right	right	dist. forearm	Machine accident
	7	21	21	22	1	right	right	wrist	Explosion injury
	Mean/SD	**26.29 ± 4.15**	**26.29 ± 4.15**	**29.14 ± 5.27**	**3.00 ± 2.89**				

The mean patient age in Group 2 (n = 7) at the time of prosthetic fitting was 26.29 ± 4.15 years (Table 1). Unilateral forearm amputations were due to high-voltage burn or industrial accidents at the level of the wrist (n = 2), distal forearm (n = 1), mid forearm (n = 3) and proximal forearm (n = 1). All prosthetic patients of this study communicated high satisfaction with their prosthetic fitting and refused to have a hand transplant mostly because of immunosuppressive drugs. Functional assessment of these male patients took place 3.00 ± 2.89 years after final fitting. Patients wore their prosthesis for an average of 8 to 16 hours per day.

### Functional Outcome

The transplanted patients achieved a mean ARAT score of 40.86 ± 8.07. The average SHAP score in this group was 75.00 ± 11.06 and the DASH score showed an average of 22.50 ± 19.73. Prosthetic patients achieved a mean ARAT score of 39.00 ± 3.61. The average SHAP score was 75.43 ± 10.81 and the DASH score showed an average of 10.83 ± 6.40 (S1 Video, Table 2).

**Table 2:** Functional outcomes.

	Hand	Patient	Side	DASH	ARAT	ARAT healthy	ARAT percent of healthy hand	SHAP	SHAP healthy	SHAP percent of healthy hand
**Group 1 Transplant**	1	1	right	27.00	50	bilateral	bilateral	84	bilateral	bilateral
	2		left	27.00	42	bilateral	bilateral	75	bilateral	bilateral
	3	2	right	55.00	31	bilateral	bilateral	72	bilateral	bilateral
	4		left	55.00	33	bilateral	bilateral	72	bilateral	bilateral
	5	3	right	8.00	44	57	77.19%	88	99	88.89%
	6	4	right	8.33	42	57	73.68%	80	101	79.21%
	7	5	right	14.17	35	57	61.4%	54	97	55.67%
		Mean/SD		**22.5 ± 19.73**	**40.86 ± 8.07**	**57.00 ± 0**	**70.76 ± 8.29**	**75.00 ± 11.06**	**99.00 ± 2.00**	**74.59 ± 17.09**
**Group 2 Prostheses**	1	1	left	7.50	42	54	77.78%	83	99	83.84%
	2	2	left	10.00	35	57	61.40%	75	97	77.32%
	3	3	right	20.83	42	57	73.68%	78	96	81.25%
	4	4	right	17.50	38	57	66.67%	54	97	55.67%
	5	5	left	9.17	36	57	63.16%	70	99	70.71%
	6	6	right	1.67	44	57	77.19%	85	98	86.73%
	7	7	right	9.17	36	57	63.16%	83	98	84.69%
		Mean/SD		**9.83 ± 6.95**	**39.00 ± 3.61**	**56.57 ± 1.13**	**69.01 ± 7.04**	**75.43 ± 10.81**	**97.71 ± 1.11**	**77.17 ± 10.90**

There were no significant differences in ARAT (p = 0.87), SHAP (p = 0.98) or DASH (p = 0.40) between the two groups. In unilateral patients, additionally the healthy hand was tested. Overall, transplanted and prosthetic hands could achieve a score of 76.40% ± 12.01 in SHAP and 69.53% ± 7.00 in ARAT in comparison to their healthy hand.

The bilateral transplanted patients achieved a mean ARAT score of 41.25 ± 10.72 and an average SHAP score of 75.75 ± 5.68. The unilateral transplanted patients achieved a mean ARAT score of 40.33 ± 4.73 and an average SHAP score of 74.00 ± 17.78. Comparing the uni- and bilateral patients within the transplant group, aside from the DASH (p = 0.03), there is no significant difference in ARAT (p = 1.00) and SHAP (p = 0.72). The difference in DASH between uni- and bilateral patients can be explained by the nature of this questionnaire asking about bimanual tasks.

### Psychosocial Outcome

The transplant patients showed significantly higher scores in the scales “role-physical”, “vitality”, “role-emotional” and “mental health” (Table 3). However, the SF-36 revealed no difference between transplanted and prosthetic patients in the other scales of “physical functioning”, “bodily pain”, “general health” and “social functioning”.

**Table 3:** Psychosocial Outcomes of Quality of Life Assessment (SF-36).

		T-values							
	Patient	Physical functioning	Role physical	Bodily pain	General health	Vitality	Social functioning	Role-emotional	Mental health
**Group 1 Transplant**	1	53^A^	58^A^	57^A^	51^A^	**64**^AA^	57^A^	54^A^	**61**^AA^
	2	48^A^	56^A^	**37**^BA^	42^A^	57^A^	57^A^	54^A^	59^A^
	3	56^A^	56^A^	55^A^	50^A^	**60**^AA^	**26**^BA^	54^A^	**60**^AA^
	4	54^A^	53^A^	48^A^	50^A^	**65**^AA^	54^A^	53^A^	**64**^AA^
	5	44^A^	53^A^	42^A^	**39**^BA^	54^A^	54^A^	53^A^	59^A^
	Mean/SD	**51.00 ± 4.90**	**55.20 ± 2.17**	**47.80 ± 8.47**	**46.40 ± 5.50**	**60.00 ± 4.64**	**49.60 ± 13.28**	**53.60 ± 0.55**	**60.60 ± 2.07**
**Group 2 Prostheses**	1	49^A^	52^A^	50^A^	41^A^	46^A^	54^A^	53^A^	54^A^
	2	**39**^BA^	44^A^	41^A^	**35**^BA^	48^A^	55^A^	53^A^	49^A^
	3	**34**^BA^	**10**^BA^	**28**^BA^	**36**^BA^	46^A^	**39**^BA^	53^A^	54^A^
	4	44^A^	**24**^BA^	55^A^	58^A^	51^A^	54^A^	53^A^	57^A^
	5	49^A^	**10**^BA^	55^A^	53^A^	51^A^	54^A^	**36**^BA^	47^A^
	6	54^A^	53^A^	48^A^	**39**^BA^	51^A^	54^A^	53^A^	52^A^
	7	**39**^BA^	**24**^BA^	55^A^	55^A^	57^A^	54^A^	53^A^	55^A^
	Mean/SD	**44.00 ± 7.07**	**31.00 ± 18.59**	**47.43 ± 9.98**	**45.29 ± 9.71**	**50.00 ± 3.83**	**52.00 ± 5.74**	**50.57 ± 6.43**	**52.57 ± 3.51**
Mann-Whitney U test		**0.144**	**0.006**	**0.975**	**0.908**	**0.008**	**0.487**	**0.035**	**0.003**

^BA^ below average.

^A^ average.

^AA^ above average.

The scores of the transplant patients were predominantly at average levels of age-equivalent male norms. Three patients even reported superior vitality and mental health. However, the third patient of this group described a reduced social functioning and the fifth patient substandard general health. Still, these results demonstrate the overall good self-reported QOL of transplant patients and their generally high satisfaction with the functional and psychosocial outcome of their hands.

The SF-36 scores of prostheses patients demonstrated their satisfaction with their mechatronic device. Some patients of this group reported reduced physical role perception, emotional role functioning, and general health. With the exception of the third patient, the results were also predominantly at average levels compared to an age-equivalent male norm sample.

## Discussion

Hands fulfill multiple motor and sensory functions in our daily lives. When only motor function is considered, this study has shown that there is no significant difference between outcomes of hand function in the prosthetic or transplantation groups. Both provide reliable and sufficient hand function for the most relevant ADL.

To our knowledge, this is the first direct comparison of outcomes on hand function following hand transplantation versus prosthetic fitting. While previous reports state that hand transplants are superior to prostheses, these claims are without sufficient outcome evidence for below-elbow amputees, as none directly compared the two methods.[35,36] The available studies enrolled different prosthetic devices including body-powered tools and were not consistent regarding the level of amputation.[23,24] And where advantages and disadvantages of the procedures have been discussed, the lack of outcome data has hindered interpretation of what is best for patients.[21] Our results provide clinicians with a practical picture of the advantages, disadvantages and most importantly, functional and psychosocial outcomes of the two procedures for patients as recommended by experts in this field.[25]

While hand transplantation promises the benefit of better aesthetic and functional outcomes as well as social benefits, this treatment also exposes patients to additional risks.[35,37] Choosing the right treatment should be dependent on what is most beneficial for the patient with the least risk of harm. Apart from long lasting rehabilitation and inpatient treatment, the greatest risk of allotransplantation is the immunosuppressive side-effects.[12,15] All patients in this study in whom hand transplantation was performed experienced between one and seven acute episodes of rejection.[12,29,38] Treatment of rejection episodes as well as long term therapy resulted in nausea, headache, fever and edema requiring hospitalization.[29] Moreover, long lasting immunosuppression increases the risk of infection, neoplasia, metabolic disorders or organ failure.[37] Details about the immunosuppressive regime and complications of the patients included in this study are published elsewhere.[29,39] Some authors state that selected patients for hand transplantation are otherwise healthy and usually do not have comorbidities that increase the likelihood of these side-effects.[9,40] However, considering all possible complications, lifelong immunosuppression can be seen as a chronic disease of initially healthy people, characterized by its own set of risks. These factors illustrate that the risks discussed are not inconsequential and imply rigorous patient selection and on-going adherence to assessment.[41]

Alternatively, prosthesis use is notoriously challenging for activities like grooming, swimming or sleeping and an estimated 20% of upper-limb amputees reportedly do not use their prostheses.[20,42] However, it has been shown, that active use of prostheses encourages continued uptake, with lower rejection-rates after early prosthetic fitting.[20] Prostheses rejecters report discomfort, lack of functional benefit, excessive weight, repetitive need for repair and lack of sensory feedback as the main reasons for discontinuing use.[42] Importantly however, prosthetic fitting has no systemic side-effects. It delivers a quick, constant and to a great extent predictable outcome, which skilled patients may further improve.

Considering the immense sensory capacity of the hand, with all its different modalities of touch, vibration, proprioception, pain and temperature, clearly a transplanted hand is the far superior choice compared to the current generation of prostheses. Even so, in unilateral amputees, regardless of reconstructive technique, the remaining healthy hand will always have better functional capacities. Indeed, unilateral amputees with one remaining healthy hand, which becomes dominant, can usually perform up to 90% of the ADL.[43] The reconstructed hand by whatever means will always be a helping hand.[9,44,45] In general, patients with bilateral amputation suffer especially from functional impairment and loss of QOL, whereas unilateral amputees primarily report difficulties with coping and psychological issues.[12,46] Notably, even 15 years after hand transplantation, the functional results seem to be constant, although regular therapy is important to maintain the achieved capacities.[28,29]

However, as all sensory feedback has been lost in bilateral amputees, it may not be sufficient to just replace motor skills. In this scenario, the benefit of restoring sensation may outweigh the risk of lifelong immunosuppression, tilting the decision making process in favor of hand transplantation. Accordingly, as has been reported by Mathes et al.[47], bilateral below-elbow amputation is the most accepted indication for hand transplantation by hand surgeons today (78%).[47] This documents a paradigm shift away from unilateral transplantation.[15] Notably, about 22% of the worldwide transplanted hands have failed due to graft loss.[14] And where transplanted hands have been re-amputated, consecutive prosthetic fitting has been successful, especially in unilateral amputees.[48]

The population sustaining severe upper extremity injuries are predominantly young, male and heavy laborers. These patients are more likely to return to work following prosthetic fitting, than after long lasting, time consuming rehabilitation and regular follow up visits to monitor possible side-effects.[49] Therefore, patient’s employment capacities and rehabilitation potentials also have to be considered before embarking on transplantation.[49]

In terms of QOL, transplanted patients show superior outcomes in four of the eight sub scores of the SF-36. Some transplant patients were even superior to an age-equivalent male norm sample. In spite of rejection episodes, the transplant patients reported satisfying physical functioning, good general health as well as appreciable vitality and mental health. As shown in this study, the individual perceived QOL of patients with prosthetic fitting might not match their actual functional outcome. These divergent results of functional and psychosocial outcome between groups demonstrate the importance of the individual’s perspective in the decision-making process. Particularly, multidisciplinary evaluation protocols enable the identification of those most suited to transplantation or prosthetic fitting. These differences may be due to the confounding effect of comparing bilateral with unilateral amputees. As such, further work is needed to directly compare specific amputation levels and type to determine if this effect is genuine. When prostheses are capable of restoring sensation, it may be worthwhile reassessing the current advantage that transplantation offers in terms of sensorimotor control, as the effect of sensation on function is definitely underestimated in the current functional testing instruments.

An additional factor to consider in this debate is the cost of each reconstructive procedure. Due to the different insurance systems and policies, the Polish patients had no access to prostheses, therefore, no affordable option other than reconstructive transplantation. As has been shown by a cost-utility analysis, prosthetic devices provide a reliable but less expensive alternative.[50] Different financial factors have to be considered, including surgical costs, inpatient treatment, hand therapy, outpatient visits, immunosuppression and time out of employment.[51] Therefore, as has been demonstrated recently by the Swiss Health Care Association, hand transplantation was rejected as a treatment modality because of the fourfold costs of hand transplantation compared to prosthetic fitting.[52]

### Limitations of this Study

Within the relatively small population of upper limb amputees, only a limited number of patients have undergone hand transplantation. As such, comparing transplant with prosthetic patients is heavily dependent on those available to take part in a clinical study. This confounding effect means that patients with critically different disabilities were compared, which may have influenced the outcomes reported here. This is particularly true for assessing the psychosocial impact of each treatment, as clearly a bilateral amputation is a more devastating injury. Further studies would benefit from a direct comparison between bilateral amputees, unilateral amputees and likewise individual levels of amputation. When prostheses are capable of restoring sensation, it may be worthwhile reassessing the current advantage that transplantation offers in terms of sensorimotor control.

## Conclusions

Hand transplantation represents a unique method of restoring a hand both from functional—motor and sensory—and psychosocial aspects such as the restoration of bodily integrity, strength and even a sense of psychological closure related to the initial traumatic event. These factors need to be considered in the decision-making process leading to patient selection. If immunosuppression is tolerated by the patient along with post-operative rehabilitation, the overall goal of restoring like with like is best achieved with hand transplantation. Therefore, in bilateral below-elbow amputees the benefits of motor and sensory restoration may outweigh the risks of life-long immunosuppression.[9,53,54] In unilateral below-elbow amputees, a prosthesis represents a useful tool assisting the remaining limb.[43,45] Unilateral amputees are able to compensate the majority of the functional deficit using their healthy hand and a prosthesis.[12] Our results clearly show, that given the lower risks associated with prosthetic fitting, this should remain the standard treatment for upper-limb amputees, especially in unilateral cases.

Both methods of treatment may further improve within the next years. The successful induction of donor-specific tolerance would prohibit the toxicity associated with non-specific immunosuppression and may therefore eliminate the risk of chronic rejection.[55] This would result in a safe transfer of composite tissue allografts and lead to improved functional outcomes, reduced morbidity and widen the range of indication.[44] On the other hand, new pattern-recognition control algorithms could revolutionize prosthetic devices.[4,18] There is also promising research into providing sensation and tactile prosthetic feedback, but at this time, not available for clinical use.(4) Both biological and technical advances will provide new possibilities, however, future developments in prosthetic technology will have great impact on the role and indications of hand transplantations.[56]

## Supporting Information

S1 Video Functional outcome, SHAP. (MP4)

## References

[1] Meyer TM. Psychological aspects of mutilating hand injuries. Hand Clin. 2003;19: 41–9. Available: http://www.ncbi.nlm.nih.gov/pubmed/12683445. 12683445 10.1016/s0749-0712(02)00056-2

[2] Herndon JH. Composite-tissue transplantation—a new frontier. N Engl J Med. 2000;343: 503–5. 10.1056/NEJM200008173430710 10944569

[3] Lanzetta M, Nolli R, Borgonovo A, Owen ER, Dubernard JM, Kapila H, et al Hand transplantation: ethics, immunosuppression and indications. J Hand Surg Br. 2001;26: 511–6. 10.1054/jhsb.2001.0635 11884098

[4] Farina D, Aszmann O. Bionic Limbs: Clinical Reality and Academic Promises. Sci Transl Med. 2014;6: 257ps12 10.1126/scitranslmed.3010453 25298319

[5] Vilkki SK, Kotkansalo T. Present technique and long-term results of toe-to-antebrachial stump transplantation. J Plast Reconstr Aesthet Surg. 2007;60: 835–48. 10.1016/j.bjps.2007.02.018 17442647

[6] Salminger S, Roche AD, Sturma A, Mayer JA, Aszmann OC. Hand Transplantation Versus Hand Prosthetics: Pros and Cons. Curr Surg Reports. 2016;4: 8 10.1007/s40137-016-0128-3 PMC4729794 26855851

[7] Benatar D, Hudson DA. A tale of two novel transplants not done: the ethics of limb allografts. BMJ. 2002;324: 971–3. Available: http://www.pubmedcentral.nih.gov/articlerender.fcgi?artid=1122909&tool=pmcentrez&rendertype=abstract. 11964346 10.1136/bmj.324.7343.971 PMC1122909

[8] Gilbert R. Transplant is successful with a cadaver forearm. Med Trib Med News. 1964;5.

[9] Schuind F, Abramowicz D, Schneeberger S. Hand transplantation: the state-of-the-art. J Hand Surg Eur Vol. 2007;32: 2–17. 10.1016/j.jhsb.2006.09.008 17084950

[10] Dubernard JM, Owen E, Herzberg G, Lanzetta M, Martin X, Kapila H, et al Human hand allograft: report on first 6 months. Lancet. 1999;353: 1315–20. 10.1016/S0140-6736(99)02062-0 10218530

[11] Kanitakis J, Jullien D, Petruzzo P, Hakim N, Claudy A, Revillard J-P, et al Clinicopathologic features of graft rejection of the first human hand allograft. Transplantation. 2003;76: 688–93. 10.1097/01.TP.0000079458.81970.9A 12973110

[12] Hautz T, Engelhardt TO, Weissenbacher A, Kumnig M, Zelger B, Rieger M, et al World experience after more than a decade of clinical hand transplantation: update on the Innsbruck program. Hand Clin. 2011;27: 423–31, viii 10.1016/j.hcl.2011.07.004 22051384

[13] Hettiaratchy S, Butler PEM. Extending the boundaries of transplantation BMJ (Clinical research ed.). 2003 pp. 1226–1227. 10.1136/bmj.326.7401.1226 PMC1126110 12791714

[14] Shores JT, Brandacher G, Lee WPA. Hand and upper extremity transplantation: an update of outcomes in the worldwide experience. Plast Reconstr Surg. 2015;135: 351e–60e. 10.1097/PRS.0000000000000892 25401735

[15] Petruzzo P, Dubernard JM. The International Registry on Hand and Composite Tissue allotransplantation. Clin Transpl. 2011; 247–53. Available: http://www.ncbi.nlm.nih.gov/pubmed/22755418. 22755418

[16] Carty MJ, Hivelin M, Dumontier C, Talbot SG, Benjoar MD, Pribaz JJ, et al Lessons learned from simultaneous face and bilateral hand allotransplantation. Plast Reconstr Surg. 2013;132: 423–32. 10.1097/PRS.0b013e318295883d 23584623

[17] Ninkovic M, Weissenbacher A, Gabl M, Pierer G, Pratschke J, Margreiter R, et al Functional outcome after hand and forearm transplantation: what can be achieved? Hand Clin. 2011;27: 455–65, viii–ix. 10.1016/j.hcl.2011.08.005 22051387

[18] Roche AD, Rehbaum H, Farina D, Aszmann OC. Prosthetic Myoelectric Control Strategies: A Clinical Perspective. Curr Surg Reports. 2014;2: 44 10.1007/s40137-013-0044-8

[19] Salminger S, Sturma A, Herceg M, Riedl O, Bergmeister K, Aszmann OC. [Prosthetic reconstruction in high amputations of the upper extremity]. Orthopade. 2015;4 10.1007/s00132-015-3113-0 25869177

[20] Biddiss EA, Chau TT. Upper limb prosthesis use and abandonment: a survey of the last 25 years. Prosthet Orthot Int. 2007;31: 236–57. 10.1080/03093640600994581 17979010

[21] Agnew SP, Ko J, De La Garza M, Kuiken T, Dumanian G. Limb transplantation and targeted reinnervation: a practical comparison. J Reconstr Microsurg. 2012;28: 63–8. 10.1055/s-0031-1281522 21717399

[22] Jablecki J, Kaczmarzyk L, Patrzalek D, Domanasiewicz A, Chełmoński A. A detailed comparison of the functional outcome after midforearm replantations versus midforearm transplantation. Transplant Proc. 2009;41: 513–6. 10.1016/j.transproceed.2009.01.006 19328915

[23] Graham B, Adkins P, Tsai TM, Firrell J, Breidenbach WC. Major replantation versus revision amputation and prosthetic fitting in the upper extremity: a late functional outcomes study. J Hand Surg Am. 1998;23: 783–91. Available: http://www.ncbi.nlm.nih.gov/pubmed/9763250. 9763250 10.1016/s0363-5023(98)80151-2

[24] Peacock K, Tsai TM. Comparison of functional results of replantation versus prosthesis in a patient with bilateral arm amputation. Clin Orthop Relat Res. 1987; 153–9. Available: http://www.ncbi.nlm.nih.gov/pubmed/3791737. 3791737

[25] Kay S, Wilks D. Invited comment: Vascularized composite allotransplantation: an update on medical and surgical progress and remaining challenges. J Plast Reconstr Aesthet Surg. 2013;66: 1456–7. 10.1016/j.bjps.2013.04.065 23972392

[26] Lyle RC. A performance test for assessment of upper limb function in physical rehabilitation treatment and research. Int J Rehabil Res. 1981;4: 483–92. Available: http://www.ncbi.nlm.nih.gov/pubmed/7333761. 7333761 10.1097/00004356-198112000-00001

[27] Yozbatiran N, Der-Yeghiaian L, Cramer SC. A standardized approach to performing the action research arm test. Neurorehabil Neural Repair. 22: 78–90. 10.1177/1545968307305353 17704352

[28] Bernardon L, Gazarian A, Petruzzo P, Packham T, Guillot M, Guigal V, et al Bilateral hand transplantation: Functional benefits assessment in five patients with a mean follow-up of 7.6 years (range 4–13 years). J Plast Reconstr Aesthet Surg. 2015;68: 1171–83. 10.1016/j.bjps.2015.07.007 26297387

[29] Weissenbacher A, Hautz T, Pierer G, Ninkovic M, Zelger BG, Zelger B, et al Hand Transplantation in Its Fourteenth Year: The Innsbruck Experience. Vasc Compos Allotransplantation. 2014;1: 11–21. 10.4161/23723505.2014.973798

[30] Kyberd PJ, Murgia A, Gasson M, Tjerks T, Metcalf C, Chappell PH, et al Case studies to demonstrate the range of applications of the Southampton Hand Assessment Procedure Practice evaluation. 2009;72: 212–218.

[31] Hudak PL, Amadio PC, Bombardier C. Development of an upper extremity outcome measure: the DASH (disabilities of the arm, shoulder and hand) [corrected]. The Upper Extremity Collaborative Group (UECG). Am J Ind Med. 1996;29: 602–8. 10.1002/(SICI)1097-0274(199606)29:6<602::AID-AJIM4>3.0.CO;2-L 8773720

[32] Turner-Bowker D, Bartley P, Ware J. SF-36^®^ Health Survey & “SF” Bibliography: Third Edition. Lincoln, QualityMetric Incorporated; 2002.

[33] Ware J, Snow K, Kosinski M, Gandek B. SF-36^®^ Health Survey Manual and Interpretation Guide. Boston: New England Medical Center, The Health Institute; 1993.

[34] Jabłecki J. World experience after more than a decade of clinical hand transplantation: update on the Polish program. Hand Clin. 2011;27: 433–42, viii 10.1016/j.hcl.2011.08.003 22051385

[35] Simmons PD. Ethical considerations in composite tissue allotransplantation. Microsurgery. 2000;20: 458–65. Available: http://www.ncbi.nlm.nih.gov/pubmed/11151000. 11151000 10.1002/1098-2752(2000)20:8<458::aid-micr19>3.0.co;2-g

[36] Schneeberger S, Ninkovic M, Gabl M, Hussl H, Rieger M, Loescher W, et al First forearm transplantation: outcome at 3 years. Am J Transplant. 2007;7: 1753–62. 10.1111/j.1600-6143.2007.01837.x 17511764

[37] Brenner MJ, Tung TH, Jensen JN, Mackinnon SE. The spectrum of complications of immunosuppression: is the time right for hand transplantation? J Bone Joint Surg Am. 2002;84-A: 1861–70. Available: http://www.ncbi.nlm.nih.gov/pubmed/12377920. 12377920

[38] Jabłecki J. World experience after more than a decade of clinical hand transplantation: update on the Polish program. Hand Clin. 2011;27: 433–42, viii 10.1016/j.hcl.2011.08.003 22051385

[39] Chełmoński A, Kowal K, Jabłecki J. The Physical and Psychosocial Benefits of Upper-Limb Transplantation: A Case Series of 5 Polish Patients. Ann Transplant. 2015;20: 639–48. Available: http://www.ncbi.nlm.nih.gov/pubmed/26502835. 26502835 10.12659/aot.893752

[40] Breidenbach WC, Tobin GR, Gorantla VS, Gonzalez RN, Granger DK. A position statement in support of hand transplantation. J Hand Surg Am. 2002;27: 760–70. Available: http://www.ncbi.nlm.nih.gov/pubmed/12239664. 12239664 10.1053/jhsu.2002.35306

[41] Schneeberger S, Landin L, Jableki J, Butler P, Hoehnke C, Brandacher G, et al Achievements and challenges in composite tissue allotransplantation Transplant International. 2011 pp. 760–769. 10.1111/j.1432-2277.2011.01261.x 21554424

[42] Biddiss E, Chau T. Upper-limb prosthetics: critical factors in device abandonment. Am J Phys Med Rehabil. 2007;86: 977–87. 10.1097/PHM.0b013e3181587f6c 18090439

[43] Bhaskaranand K, Bhat AK, Acharya KN. Prosthetic rehabilitation in traumatic upper limb amputees (an Indian perspective). Arch Orthop Trauma Surg. 2003;123: 363–6. 10.1007/s00402-003-0546-4 12827395

[44] Piza-Katzer H, Wechselberger G, Estermann D, Gabl M, Arora R, Hussl H. [Ten years of hand transplantation experiment or routine?]. Handchirurgie, Mikrochirurgie, Plast Chir Organ der Deutschsprachigen Arbeitsgemeinschaft für Handchirurgie Organ der Deutschsprachigen Arbeitsgemeinschaft für Mikrochirurgie der Peripher Nerven und Gefässe Organ der Vereinigung der Deut. 2009;41: 210–6. 10.1055/s-0029-1225628 19688651

[45] Aszmann OC, Roche AD, Salminger S, Paternostro-Sluga T, Herceg M, Sturma A, et al Bionic reconstruction to restore hand function after brachial plexus injury: a case series of three patients. Lancet. 2015; 10.1016/S0140-6736(14)61776-1 25724529

[46] Kumnig M, Jowsey SG, Rumpold G, Weissenbacher A, Hautz T, Engelhardt TO, et al The psychological assessment of candidates for reconstructive hand transplantation. Transpl Int. 2012;25: 573–85. 10.1111/j.1432-2277.2012.01463.x 22448727

[47] Mathes DW, Schlenker R, Ploplys E, Vedder N. A survey of north american hand surgeons on their current attitudes toward hand transplantation. J Hand Surg Am. 34: 808–14. 10.1016/j.jhsa.2009.01.021 19410983

[48] Unger L. Louisville doctors remove transplanted hand. The Courier-Journal. 2014 Available: http://www.courier-journal.com/story/news/local/2014/12/01/louisville-doctors-remove-transplanted-hand/19753277/.

[49] Pinzur MS. Amputations and prosthetics. Chir Narzadow Ruchu Ortop Pol. 1999;64: 571–81. Available: http://www.ncbi.nlm.nih.gov/pubmed/10676021. 10676021

[50] Chung KC, Oda T, Saddawi-Konefka D, Shauver MJ. An economic analysis of hand transplantation in the United States. Plast Reconstr Surg. 2010;125: 589–98. 10.1097/PRS.0b013e3181c82eb6 19910847 PMC4387885

[51] Chang J, Mathes DW. Ethical, financial, and policy considerations in hand transplantation. Hand Clin. 2011;27: 553–60, xi 10.1016/j.hcl.2011.07.006 22051396

[52] Brügger U. Schould hand transplantation be reimbursed by a public payer? An up-to-date of a Swiss HTA for decision making in health care. HTAi Conference, Seoul. 2013.

[53] Piza-Katzer H. My reflections and opinions on hand transplantation. J Hand Surg Br. 2001;26: 518 10.1054/jhsb.2001.0672 11884100

[54] Piza-Katzer H, Ninkovic M, Pechlaner S, Gabl M, Hussl H. Double hand transplantation: functional outcome after 18 months. J Hand Surg Br. 2002;27: 385–90. Available: http://www.ncbi.nlm.nih.gov/pubmed/12162985. 12162985 10.1054/jhsb.2002.0759

[55] Szajerka T, Klimczak A, Jablecki J. Chimerism in hand transplantation. Ann Transplant. 16: 83–9. Available: http://www.ncbi.nlm.nih.gov/pubmed/21436781. 21436781

[56] Jones NF. Concerns about human hand transplantation in the 21st century. J Hand Surg Am. 2002;27: 771–87. Available: http://www.ncbi.nlm.nih.gov/pubmed/12239665. 12239665 10.1053/jhsu.2002.34373

